# The Impact of Hypotensive Epidural Anesthesia on Distal and Proximal Tissue Perfusion in Patients Undergoing Total Hip Arthroplasty

**DOI:** 10.4172/2155-6148.1000366

**Published:** 2013-11-29

**Authors:** Thomas Danninger, Ottokar Stundner, Yan Ma, James J Bae, Stavros G Memtsoudis

**Affiliations:** 1 Department of Anesthesiology, Hospital for Special Surgery, Weill Cornell Medical College, New York, NY, USA; 2 Department of Anesthesiology, Perioperative Medicine and Intensive Care Medicine, Paracelsus Medical University, Salzburg, Austria

## Abstract

Little data exists to detail the effect of hypotensive epidural anesthesia on differential tissue oxygenation changes above and below the level of neuraxial blockade. This study was designed to investigate tissue oxygenation in a clinical setting, using non-invasive near-infrared spectroscopy.

**Methods:**

Patients aged 18 to 85 years scheduled to undergo primary total hip arthroplasty were enrolled. Muscle oxygenation saturation was measured above and below the level of neuraxial blockade (deltoid and vastus lateralis muscles). Other continuously recorded parameters included cardiac output, stroke volume, heart rate, invasive mean arterial blood pressure and arterial oxygen saturation. Recordings of muscle oxygenation were compared over time separately for upper and lower extremity.

**Results:**

10 patients were enrolled. We found an intermittent and significant unadjusted decline of mean muscle oxygenation saturation in the vastus lateralis muscle during first part of the surgery (nadir 2^nd^ quintile: 71.0% vs. 63.3%, p<0.0001). This decline was followed by a return to baseline towards the end of the surgery (71.0% vs. 69.1%, p=0.3429). Mean muscle oxygenation saturation did not change for the same period of time in the deltoid muscle. When adjusting for covariates, the changes in muscle tissue oxygenation remained significant.

**Conclusion:**

These results indicate that muscle oxygenation saturation, a surrogate parameter for tissue perfusion, is decreased by hypotensive epidural anesthesia, but only within the functional limits of the neuraxial blockade. The etiology of these findings remains to be elucidated.

## Introduction

Total hip arthroplasties (THA) are performed more than 280,000 times per year in the United States with predictions for this number to more than triple within 20 years [1,2]. Increasing evidence suggests that neuraxial anesthesia may be associated with better outcomes as compared to general anesthesia [3,4], especially in regard to perioperative blood loss and risk for thromboembolic events. Although the mechanisms by which this effect is achieved are not fully understood, data suggest that neuraxial anesthesia induced sympatholysis is a major contributor [4,5].

In an attempt to enhance these beneficial effects and based on the theory of reduction of blood pressure while maintaining flow via stabilization of cardiac output (CO), the technique of hypotensive epidural anesthesia (HEA) was developed decades ago and since used with great success [6–11]. This technique is especially useful in patients undergoing THA where blood supply to the operative site cannot be reduced by tourniquet inflation as commonly done in total knee arthroplasties [12].

However, little data exists to detail the effect of hypotensive epidural anesthesia on tissue perfusion above and below the level of sympatholysis. Therefore, we sought to measure muscle tissue oxygen saturation (SmO_2_) as a marker of tissue perfusion above and below the level of sympatholysis under conditions of HEA in THA patients [13]. Specifically we sought to elucidate changes over time in SmO_2_ at the deltoid and the vastus lateralis muscle.

We hypothesized that we would find no difference between SmO_2_ measured at the deltoid versus the vastus lateralis muscle in patients undergoing THA under HEA.

## Material and Methods

### Ethics approval, demographics

After approval by the institutional review board, patients aged 18 to 85 years undergoing THA under HEA were enrolled. Data on patient demographics (age, gender, ethnicity, comorbidities) and intraoperative events (fluid balance, duration of surgery) were recorded.

### Data collection

SmO_2_ was measured by continuous sampling of non-invasive near-infrared spectroscopy (NIRS) at two standardized locations above and below the level of neuraxial blockade ((a) deltoid and (b) vastus lateralis of the quadriceps femoris muscle) using two CareGuide^™^ NIRS devices (Reflectance Medical, Westborough, MA). Stroke volume (SV) and CO were recorded continuously using a validated non-invasive bioreactance monitor (NICOM^™^; Cheetah Medical, Vancouver, WA). Other continuously recorded parameters included heart rate (HR), invasive mean arterial pressure (MAP) and arterial oxygen saturation (SO_2_). The observation period was divided into quintiles to facilitate analysis.

### Combined spinal epidural anesthesia

All patients were under the care of one anesthesiologist experienced in HEA. After establishing monitoring according to the ASA standards, additional monitoring for the study was applied as described above. Combined spinal/epidural anesthesia (CSE) was administered using bupivacaine 0.5% 2.5 cc intrathecally. For sedation purposes midazolam 5 mg and adequately titrated propofol to achieve sedation but maintain spontaneous respiration were used. Epidurally administered lidocaine 2% in 5 ml aliquots was used to achieve a mid thoracic sympathetic level resulting in hypotension (goal: 50–55 mmHg). For the purpose of maintaining CO, normovolemia was targeted and a low dose epinephrine intravenous infusion was used as per institutional HEA protocol^1^.

### Statistics

The primary outcome of interest was a change in SmO_2_ and secondary outcomes include changes in CO, HR, and MAP. In order to examine the changes in these outcomes over time, we modeled the outcomes as functions of time (i.e., nadir quintiles) using linear regression with inference based on the generalized estimating equations (GEE) method [14,15]. The GEE method is able to take into account correlations between repeated measures and has been recommended for analysis of longitudinal data in a variety of research including anesthesia study [14]. Specifically, we first ran unadjusted regression analyses where time is the only covariate. We then ran adjusted regression analyses to control for patient demographics (age, gender, BMI) when assessing changes over time for each outcome. In addition to patient demographics, CO, and location of measurement (deltoid and thigh) were adjusted simultaneously for SmO_2_.

## Results

We enrolled a total of 10 patients undergoing THA under HEA in this study; patient’s demographics and mean procedure time are shown in Table 1. Notably, no patients had significant cardiopulmonary disease and only four cases in, whom hypertension, hyperlipidemia or rheumatoid arthritis was present were, recorded.

**Figure 1:**
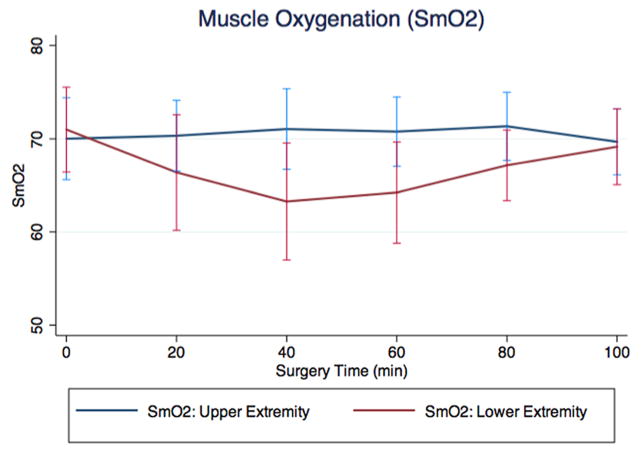
Muscle oxygenation (SmO_2_) for upper (deltoid muscle) and lower extremity (vastus lateralis muscle). This figure depicts the decline in SmO_2_ in the lower extremity (vastus lateralis muscle, red line) in contrast to SmO_2_ (%) in the upper extremity (deltoid muscle, blue line).

From the unadjusted regression analyses we found an intermittent and significant decline of mean SmO_2_ in the vastus lateralis muscle during the first part of the surgical procedure in patients undergoing THA using HEA (nadir 2^nd^ quintile: 71.0% vs. 63.3%, p<0.0001). This decline was followed by a return to baseline towards the end of the surgery (71.0% vs. 69.1%, p=0.3429). In contrast, mean SmO_2_ did not change for the same period of time in the deltoid muscle (Table 2 and Figure 1). When analyzing vital parameters, we found a significant decline of MAP during surgery (nadir 2^nd^ quintile) followed by a return to the initial values (65.5 (SD ± 14.2) vs. 56.6 [(SD ± 5.7) p=0.011] and 65.5 (SD ± 14.2) vs. 66.1 mmHg [(SD ± 7.3), p=0.903], respectively,); mean HR showed a modest but significant increase over the same period (66.2 (SD ± 9.1) vs. 71.5 [(SD ± 7.6). p=0.005] and 66.2 (SD ± 9.1) vs. 74.6 bpm [(SD ± 7.6) p=0.0001], respectively). No significant change was seen for mean CO over time (3.51 (SD ± 0.66) vs. 3.78 [(SD ± 0.82) p=0.2565] and 3.51 (SD ± 0.66) vs. 4.05 L/min [(SD ± 1.20) p=0.0515], respectively). Mean values for each quintile of parameters obtained are shown in Table 3.

In the adjusted regression analyses, significant changes in SmO_2_ with respect to baseline were identified for the first part of the surgical procedure (Table 2). There was no significant influence of other variables on SmO_2_.

## Discussion

In this study evaluating changes in SmO_2_ above and below the level of sympathetic blockade in patients undergoing THA under HEA, we found a significant decline of SmO_2_ of the vastus lateralis muscle but not the deltoid, suggesting differential effects on tissue perfusion for the different locations of measurement.

This decrease in SmO_2_ in the vastus lateralis muscle was surprising, as we expected that perfusion would remain unaffected in the area of sympathectomy. Although reasons for this finding will have to remain speculative at this point, they include the following possibilities: NIRS equipment was attached to the dependent thigh as to avoid interfering with surgery and in the assumption that external manipulation of the area would be minimal. However, compression of blood vessels may still occur during the surgical procedure by positioning posts or due to kinking of vessels during dislocation of the hip joint. Alternatively and in addition, dislocation of the hip and positioning may be associated with increased abdominal pressure thus impeding venous return, which in turn may decrease micro perfusion in the context of already decreased arterial pressure. All circumstances mentioned above may contribute to the observed decrease in SmO_2_ in the lower extremity. Whatever the reason, a clear correlation with changes in MAP were noticed suggesting significant influence of driving pressures in large arteries on measurements.

SmO_2_ was maintained in the deltoid despite a reduction in MAP. While not affected by the sympathectomy of the neuraxial blockade, this finding suggests that the interventions applied to achieve HEA also preserve above the level of blockade.

As previously described we found a significant decrease in MAP while CO was maintained in line with the concept of HEA, which seeks to maintain perfusion at lower pressures by maintaining cardiac filling and contractility. Thus our results suggest that concerns of hypoperfusion of levels above the sympathetic block during HEA are unfounded, at least in the context of our practice.

Some limitations to this study have to be addressed. First, this clinical trial has been designed as a proof of concept study to obtain information for larger upcoming clinical investigations. Thus the number of patients included to the study is relatively small and may impact on the outcomes, particularly as analyzed using multivariate analysis. Therefore, conclusions from the results of this study have to be drawn carefully. Second, the NIRS device was validated using a setting of lower body negative pressure (LBNP) which cannot directly be extrapolated to our setting [13,16]. Nevertheless, muscle tissue oxygenation has been shown to be a marker of tissue perfusion in the referred model. Third, there may be more direct approaches to measure tissue perfusion [17], however the use of this device allowed for non-invasive measurement thus minimizing patient discomfort and risk of injury [18].

In conclusion, this study showed a significant unadjusted decrease of muscle tissue oxygenation saturation of the vastus lateralis muscle in patients undergoing THA using HEA. The SmO_2_ in the deltoid muscle remained unaffected. Except changes in MAP, no other studied covariates impacted significantly on outcomes. Reasons for the former finding have to remain speculative. While correlation with actual clinical outcomes is warranted, the maintenance of SmO_2_ in the deltoid is encouraging and suggests adequate tissue perfusion to the level above the sympathetic block. Therefore, further research with a larger number of patients is needed to determine if these findings are of clinical significance.

## Figures and Tables

**Table 1:** Patient related demographics. Data is presented as mean and standard deviation (SD).

Patient related demographics	
Average age (years)	60.6 (12.5)
BMI kg/m^2^	26.23 (5.63)
Sex (female/male)	7/3
Procedure time (min)	73.3 (15.8)
Intraoperative fluid (mL)	1528 (326)

**Table 2:** Change in SmO_2_ against baseline (unadjusted and adjusted analysis) for upper (deltoid muscle) and lower extremity (vastus lateralis muscle). This table displays the unadjusted (deltoid and vastus lateralis muscle) and adjusted analysis of SmO_2_ for each quintile (SmO_2_ presented as means plus standard deviation (SD); q0 represents baseline; qX: quintile 0–5).

Change in SmO_2_ (in %, unadjusted analysis)						Change in SmO_2_ (in %, adjusted analysis)		
Deltoid (SD)			Vastus lateralis (SD)					
q0	70.01 (8.81)	**p-value**	q0	70.99 (9.11)	**p-value**	q0	69.93 (5.47)	**p-value**
q1	70.33 (7.61)	0.7534	q1	66.38 (12.41)	0.0105	q1	67.74 (6.45)	0.0294
q2	71.05 (8.65)	0.5295	q2	63.27 (12.57)	<.0001	q2	66.45 (6.54)	0.0032
q3	70.77 (7.45)	0.6754	q3	64.23 (10.88)	0.0001	q3	66.79 (6.54)	0.0081
q4	71.34 (7.33)	0.4826	q4	67.15 (7.59)	0.0197	q4	68.49 (5.44)	0.3315
q5	69.68 (7.09)	0.8736	q5	69.15 (8.14)	0.3429	q5	68.57 (5.50)	0.4366

**Table 3:** Vital parameters and muscle tissue oxygen saturation (SmO_2_) for upper (deltoid muscle) and lower extremity (vastus lateralis muscle). This table displays the means (plus standard deviation) for vital parameters and muscle tissue oxygen saturation for each quintile. (q0 represents baseline; qX: quintile 0–5).

	SmO_2_ [Deltoid, %]	SmO_2_ [Vastus lateralis, %]	HR [bpm]	Psys [mmHG]	Pdia [mmHG]	MAP [mmHG]	SpO_2_ [%]	CO [L/min]
**q0**	70.01 (8.81)	70.99 (9.11)	66.17 (9.07)	89.00 (20.90)	51.36 (11.29)	65.53 (14.24)	97.79 (2.19)	3.51 (0.66)
**q1**	70.33 (7.61)	66.38 (12.41)	68.19 (6.75)	80.47 (14.19)	46.74 (8.42)	58.78 (10.19)	97.65 (2.10)	3.60 (0.69)
**q2**	71.05 (8.65)	63.27 (12.57)	71.75 (7.64)	77.74 (8.25)	45.28 (5.41)	56.60 (5.70)	97.81 (2.03)	3.78 (0.82)
**q3**	70.77 (7.45)	64.23 (10.88)	72.03 (6.64)	80.11 (6.98)	45.80 (5.57)	57.67 (4.63)	97.97 (1.70)	3.77 (0.87)
**q4**	71.34 (7.33)	67.15 (7.59)	74.96 (5.28)	84.51 (7.90)	47.21 (3.86)	59.81 (2.73)	98.27 (1.44)	3.87 (1.00)
**q5**	69.68 (7.09)	69.15 (8.14)	74.61 (7.61)	95.42 (14.76)	50.83 (4.56)	66.14 (7.27)	98.45 (1.75)	4.05 (1.20)

## Footnotes

1 http://hss.edu/anesthesiology-pro-hypotensive-epidural-anesthesia.asp.
